# Targeted sequencing provides genetic insights into familial cardiovascular disease among young adults in a high consanguinity population

**DOI:** 10.1371/journal.pone.0353861

**Published:** 2026-08-13

**Authors:** Nauman Arif, Imran Khan, Taj Ali Khan, Sajjad Ahmad, Fatima Nauman, Gulzar Ahmad, Muhammad Ijaz, Bilal Ahmad, Syed Munawar Hassan, Saima Afaq, Zia Ul Haq

**Affiliations:** 1 Institute of Public Health and Social Sciences, Khyber Medical University, Peshawar, Pakistan; 2 Department of Cardiology, Lady Reading Hospital, Peshawar, Pakistan; 3 Institute of Pathology and Diagnostic Medicine, Khyber Medical University, Peshawar, Pakistan; 4 Department of General Surgery, Prime Teaching Hospital, Peshawar, Pakistan; 5 Office of Research Innovation and Commercialization, Khyber Medical University, Peshawar, Pakistan; 6 Department of Health Sciences, University of York, York, United Kingdom; The University of Texas Rio Grande Valley, UNITED STATES OF AMERICA

## Abstract

**Background:**

Cardiovascular diseases (CVDs) account for approximately 19.8 million deaths annually, with coronary artery disease (CAD) as a major contributor. Genetic factors play an important role in CVD development. Genetic risk can be attributed to monogenic and polygenic risk variants. Patients with rare, Mendelian monogenic CVDs have been shown to have mutations in many causative genes, including *LRP6, MEF2A, CYP27A1,* and *ST6GALNAC5.*

**Methods:**

A case-control study was conducted at two tertiary care hospitals in Peshawar, Pakistan, Lady Reading Hospital and Peshawar Institute of Cardiology. The study included 20 patients with familial CVDs diagnosed through clinical and laboratory investigations and 20 matched healthy controls. Whole blood and relevant clinical-demographic data were collected, with matched control samples selected by age, gender, and family background, with minimizing variability to ensure accurate comparison. A total of 5 *mL* of blood was drawn from each patient and transported to the Molecular and Genomic Laboratory (IPDM) at Khyber Medical University. DNA was extracted from the collected samples, and targeted PCR and Sanger sequencing were performed. The sequencing data were analyzed using bioinformatics tools such as FinchTV and BioEdit.

**Results:**

The analysis demonstrated the presence of mutations in 12 out of 20 patient’s DNA, including *LRP6* gene variants (146344T > A in two patients, 146345A > G in three, and 92497C > A and 94776T > A in one each), *MEF2A* gene variants (113826A > T in one patient and 113802T > A in two), and *CYP27A1* gene variants (5924G > C and 6016G > A in one patient each).

**Conclusion:**

This study identified genetic variants in the *LRP6*, *MEF2A*, and *CYP27A1* genes among young patients with familial CVD using targeted PCR and Sanger sequencing. The detection of these variants in a proportion of cases suggests a possible genetic contribution within the studied cohort. However, further studies with larger sample sizes and advanced sequencing approaches (WGS/NGS) are required to confirm the clinical significance of these findings.

## Introduction

Cardiovascular diseases (CVDs) are the leading cause of mortality globally, accounting for approximately 19.8 million deaths annually, or 32% of all global deaths. Among these, ischemic heart disease and stroke remain the most significant contributors. The burden of CVDs varies across regions and populations, influenced by socioeconomic disparities, healthcare infrastructure, access to preventive services, and underlying genetic and lifestyle factors [1,2]. In low and middle income countries (LMICs), the CVD burden is disproportionately higher due to limited access to quality healthcare, late presentation to hospitals, and an increased prevalence of modifiable risk factors including hypertension, obesity, smoking, and diabetes [3]. In Pakistan, the age-standardized incidence rate of CVDs is estimated at 918.18 per 100,000 populations, which surpasses the global average of 684.33 per 100,000 [4]. The age-standardized death rate from CVDs in Pakistan is 357.88 per 100,000, also significantly exceeding the global average of 239.85 per 100,000 [5]. While traditional risk factors such as tobacco use, physical inactivity, unhealthy diet, and obesity remain significant, genetic predisposition plays an increasingly recognized role in CVD susceptibility [2,6]. Family history of premature coronary artery disease (CAD) is associated with a two-fold increase in risk, independent of lifestyle-related factors [7]. Heritability estimates for CAD range between 40% and 60% based on family-based and twin studies, particularly in early-onset cases [8]. Advances in genomic epidemiology and high-throughput sequencing technologies have revolutionized our understanding of genetic contributions to CVD, allowing for the identification of numerous risk loci through genome-wide association studies (GWASs) [9,10]. A landmark discovery in 2007 identified the 9p 21.3 locus as a significant contributor to CAD risk, independent of conventional risk factors [11]. This 53-kb region is highly conserved and confers a ~ 40% increased risk for CAD, especially in homozygous individuals [12]. Large consortia such as CARDIOGRAM and C4D have subsequently expanded the list of CAD-associated loci, identifying more than 160 common variants that contribute cumulatively to disease risk [13,14]. However, most of these variants confer only modest effect sizes (OR < 1.15), suggesting the need to explore rare genetic variants that may explain the so called missing heritability in CVD [15,16]. Recent advances in next-generation sequencing (NGS) have facilitated the discovery of both common and rare variants that influence CVD risk [17]. NGS technologies have significantly reduced the cost and time required to sequence entire genomes, allowing for more comprehensive exploration of both monogenic and complex polygenic forms of CVD [18]. Rare variant analysis, as part of the NHLBI Exome Sequencing Project and 1000 Genomes Project, has identified pathogenic mutations that are particularly relevant in early-onset or familial cases of CVD [19]. Among the key genes of interest in CVD genetics is *LRP6* (Low-Density Lipoprotein Receptor-Related Protein 6), a critical co-receptor in the Wnt signaling pathway [20,21]. This pathway regulates essential processes such as cellular proliferation, differentiation, and tissue maintenance. Aberrant Wnt signaling, mediated through *LRP6* dysfunction, has been implicated in a wide spectrum of cardiovascular pathologies including arrhythmias and structural heart defects [22]. *LRP6* is prominently localized at cardiac gap junctions and contributes to normal electrical conduction and cardiac rhythm. Its dysfunction can compromise intercellular communication and predispose individuals to arrhythmias and cardiomyopathies [23]. Another important gene in cardiovascular remodeling is *MEF2A* (Myocyte Enhancer Factor 2A), a transcription factor involved in cardiac development, endothelial function, and hypertrophy signaling [24]. A 21-base pair deletion in exon 11 of *MEF2A* was first reported in a Scandinavian family with early-onset CAD and myocardial infarction (MI), disrupting nuclear localization and transcriptional activity [25]. Functional variants in exon 7 (e.g., Asn263Ser, Pro279Leu, and Gly283Asp) have also been identified exclusively in affected individuals, reinforcing its role in endothelial dysfunction and atherosclerosis [26]. *MEF2A* integrates signals from key hypertrophic pathways including CaMKII*,* MAPK*,* and PI3K/Akt*,* with post-translational modifications such as phosphorylation and SUMOylation modulating its transcriptional activity [27]. Although *MEF2A* was initially proposed as a causative gene for CAD, it has not consistently emerged as a significant risk locus in large-scale GWASs. However, the highly polymorphic nature of exon 11, including the (CAG)n repeat region, continues to be investigated in various populations [28]. In hypertensive hearts, *MEF2A* expression is upregulated in response to pressure overload, contributing to pathological cardiac remodeling and heart failure [29]. CVD remain a major public health concern, particularly in younger populations in developing regions. Genetic factors play an important role in disease susceptibility, especially in familial cases. However, there is limited data on genetic variants in Pakistani high-consanguinity populations. Therefore, the present study was designed to explore pathogenic variants in selected CVD related genes, including *LRP6, MEF2A, CYP27A1,* and *ST6GALNAC5,* among patients with familial cardiovascular disease in Peshawar Khyber Pakhtunkhwa, Pakistan, with the aim of improving insight into the genetic basis of early-onset and inherited CVDs and informing targeted preventive approaches.

## Materials and methods

### Study design and setting

This case-control study was conducted at two tertiary care hospitals in Peshawar, Pakistan, Lady Reading Hospital and the Peshawar Institute of Cardiology. All data collection procedures were conducted in accordance with established ethical research standards and approved protocols. The study involved 20 clinically diagnosed familial CVD cases and their matched controls from the same families, ensuring genetic and demographic comparability. The study recruited patients over a six-month period, from 05 September 2024**–**05 March 2025**.**

### Ethical considerations

This study was conducted in accordance with the ethical principles outlined in the Declaration of Helsinki. The study protocol received ethical clearance from the Advanced Studies and Research Board (ASRB) and the Ethical Review Committee of Khyber Medical University. Written informed consent was obtained from all participants following a thorough explanation of study objectives, risks, and their right to withdraw at any stage. Confidentiality and voluntary participation were emphasized throughout the process.

### Data and sample collection

Demographic data (age, sex, occupation, education, residential status, and disease duration) were recorded using a structured proforma. Peripheral venous blood (5 mL) was collected under sterile conditions in EDTA tubes by a trained phlebotomist and transported to the Molecular and Genomic Laboratory, Institute of Pathology and Diagnostic Medicine (IPDM), KMU, for further analysis.

### DNA extraction

Genomic DNA was isolated from whole blood using a standard lysis and column-based purification protocol. Briefly, red blood cells were lysed, followed by white blood cell lysis using Proteinase K (Sigma-Aldrich) and ethanol (Sigma-Aldrich) precipitation. DNA was purified using spin columns (Thermo Fisher Scientific) and eluted in pre-warmed elution buffer (Thermo Fisher Scientific). DNA quality and quantity were assessed via agarose gel electrophoresis and NanoDrop (Thermo Fisher Scientific).

### DNA quantification

A 1% Agarose gel (Sigma-Aldrich) stained with Ethidium bromide (Thermo Fisher Scientific) was used to verify DNA integrity. For quantitative assessment, DNA absorbance was measured at 260 nm using a NanoDrop spectrophotometer (Thermo Fisher Scientific). Purity was determined by evaluating A260/A280 and A260/A230 ratios, with values close to 1.8 indicating acceptable purity.

### Primer design

Primers for target genes (*LRP6*, *MEF2A*, *CYP27A1*, and *ST6GALNAC5*) were designed using Primer 3 [30] and NCBI Primer–BLAST [31]. Design parameters included primer lengths of 18–22 bp, melting temperatures of 55–65°C, GC content of 40–60%, minimal secondary structures, and expected amplicon sizes of 650–900 bp. Specificity was verified using BLAST. Result were also shown in Table 1.

**Table 1:** Primer sequences used for PCR amplification.

Gene	Forward Primer (5’–3’)	Reverse Primer (5’–3’)	Product Size (bp)
*LRP6*	CCCCTTCCTTGGCATTAACT	CCCCAACACCAGTTAACAGC	799
*MEF2A*	CTGGTCTGCCACCTCAGAA	AAAACGGGGGCAGTGTGTAA	735
*CYP27A1*	CTCGACCCAAAGGTGCAG	TCCACGATCTGAGATGCAGC	770
*ST6GALNAC5*	CTAGGGGACGGTGCTTTCTC	CAACCCCAGTTTCCTCCCAC	781

### PCR amplification and product analysis

PCR was performed in 20 µL reaction mixtures using gene-specific primers, template DNA, dNTPs (Thermo Fisher Scientific), Taq polymerase (Thermo Fisher Scientific), MgCl_2_ (Thermo Fisher Scientific), and PCR buffer (Thermo Fisher Scientific). Thermal cycling included initial denaturation at 95°C for 5 minutes, followed by 40 cycles at 95°C (30 s), 58°C (30 s), and 72°C (60 s), with a final extension at 72°C for 10 minutes. A no-template control was used to detect contamination. PCR products were separated on 1% agarose gel and visualized under UV light in Gel Documentation system. Product sizes were confirmed using a specific molecular weight DNA ladder.

### Sanger sequencing and bioinformatics analysis

PCR products were purified by ethanol precipitation and sequenced using the BigDye Terminator v3.1 Sanger sequencing system (Thermo Fisher Scientific). Sequencing reactions were performed under standard thermal cycling conditions, and products were purified, denatured, and run on an ABI 3730 automated sequencer (Thermo Fisher Scientific). Sequence data were analyzed using FinchTV [32], Chromas [33], and BioEdit software [34], and compared with reference sequences using NCBI BLAST [35]. Sanger sequencing chromatograms were initially visualized and quality-checked using FinchTV. The processed sequences were then compared with reference sequences to identify genetic variants through NCBI. Briefly, identified variants were cross checked against publicly available databases including ClinVar for clinical significance and dbSNP for reference SNP identification (rs numbers). These databases were used to confirm whether the detected variants had been previously reported and to classify their potential clinical relevance.

## Results

A total of 20 clinically confirmed patients with familial cardiovascular diseases and their matched controls were included in the analysis. Genetic sequencing revealed mutations in 12 of the 20 patients. Variants were identified across multiple candidate genes associated with CVD, including *LRP6, MEF2A,* and *CYP27A1.* Specifically, *LRP6* variants were the most frequently observed, followed by mutations in *MEF2A* and *CYP27A1* and there were no mutations observed in the *ST6GALNAC5* gene.

- ***LRP6 Gene Mutations:***
  - The 146344T>A mutation was identified in two patients.
  - The 146345A>G mutation was observed in three patients.
  - The 92497C>A and 94776T>A mutations were detected in one patient each.
- ***MEF2A* Gene Mutations**:
  - A mutation at position 113826A>T was found in one patient.
  - Another mutation at position 113802T>A was detected in two patients.
- ***CYP27A1* Gene Mutations**:
  - The 5924G>C mutation was identified in one patient.
  - The 6016G>A mutation was detected in another patient.

### Mutations in *LRP6* genes

**Fig 1:**
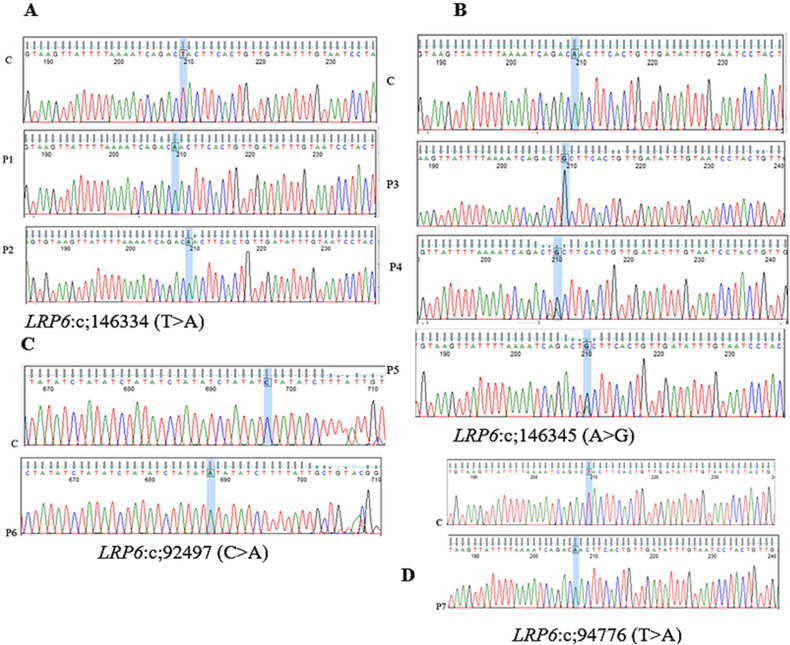
Represent Sanger sequencing chromatograms of *LRP6* gene variants. Sanger sequencing chromatograms showing sequence variants identified in the *LRP6* gene among the studied patients. **(A)** Homozygous *LRP6* c.146344T > A substitution detected in patients P1 and P2 compared with the wild-type control sequence (C). **(B)** *LRP6* c.146345A > G substitution identified in patients P3–P5, including a homozygous variant in P3 and heterozygous variants in P4 and P5, relative to the control sequence (C). **(C)** Homozygous *LRP6* c.92497C > A substitution identified in patient P6 compared with the corresponding wild-type control sequence C. **(D)** Representative chromatogram demonstrating the *LRP6* c.94776T > A sequence variant identified in patient P7 relative to the control sequence (C). Homozygous variants are characterized by complete nucleotide replacement, whereas heterozygous variants show overlapping dual peaks at the variant position.

Mutations in the *LRP6* gene were identified at several nucleotide positions across the analyzed samples. At nucleotide position c.146334 (T > A), mutations were observed in two samples (P1 and P2), both of which were homozygous. In Fig 1A, “C” represents the wild-type (control), while P1 and P2 represent the mutant samples. At nucleotide position c.146345 (A > G), mutations were detected in three samples (P3, P4, and P5). Among these, one sample (P3) exhibited a homozygous mutation, while the remaining two samples (P4 and P5) showed heterozygous mutations, as illustrated in Fig 1B, where “C” represents the wild-type control. At nucleotide position c.92497 (C > A), mutations were identified in two samples, both demonstrating homozygous alterations. In Fig 1C, “C” represents the wild-type control, while the mutant sample (P6) is shown accordingly. At nucleotide position c.94776 (T > A), a mutation was identified in one sample (P7), which was homozygous. In Fig 1D, chromatogram demonstrating the *LRP6* c.94776T > A sequence variant identified in patient P7 relative to the control sequence (C). Homozygous variants are characterized by complete nucleotide replacement, whereas heterozygous variants demonstrate overlapping dual peaks at the variant position.

### Gel electrophoresis

**Fig 2:**
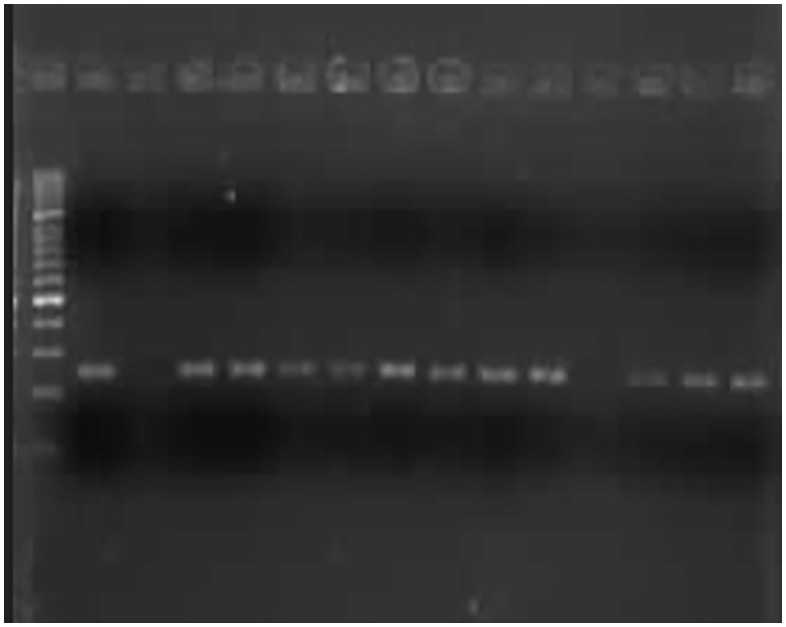
Agarose gel electrophoresis of PCR-amplified LRP6 gene fragments. Agarose gel electrophoresis showing PCR amplification of the *LRP6* gene in control and patient samples. Lane M, DNA molecular weight marker (DNA ladder); lanes P1–P7, PCR-amplified *LRP6* gene fragments from patients P1–P7. The presence of distinct bands at the expected amplicon size confirms successful amplification of the target LRP6 gene in all patient samples.

The agarose gel electrophoresis image demonstrates the successful amplification of the *LRP6* gene across all analyzed samples. The DNA ladder serves as a molecular size reference for estimating fragment lengths. The control sample shows a clear band corresponding to the expected amplicon size, confirming the validity of the PCR reaction. All patient samples (P1–P7) exhibit distinct bands at similar positions, indicating successful amplification of the target gene region. No significant variation in band size is observed among samples, suggesting that the detected mutations are point mutations rather than insertions or deletions, as such changes do not alter fragment length. The presence of single, well-defined bands without smearing indicates high-quality DNA and specific amplification. These PCR products were subsequently subjected to sequencing analysis, through which multiple nucleotide substitutions in the *LRP6* gene were identified, including: c.146334 (T > A) – homozygous mutations in P1 and P2, c.146345 (A > G) – one homozygous (P3) and two heterozygous (P4, P5) mutations, c.92497 (C > A) – homozygous mutation in P6, c.94776 (T > A) – homozygous mutation in P7. The gel confirms the integrity and suitability of PCR products for downstream sequencing, which enabled the identification of these genetic variants. As shown in Fig 2.

### Mutation in the *MEF2A* gene

**Fig 3:**
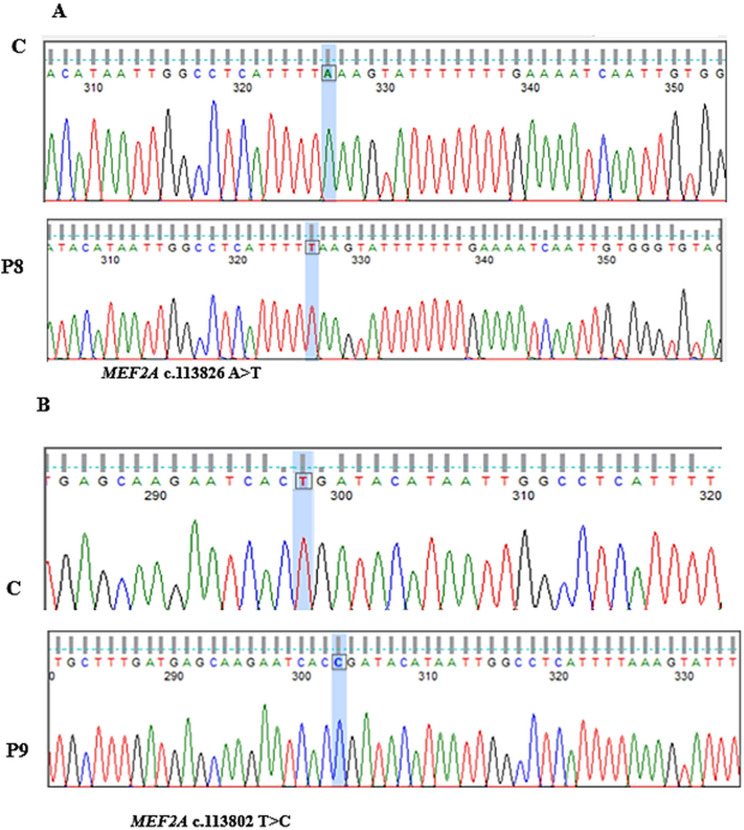
Shows sanger sequencing chromatograms of *MEF2A* gene variants. Sanger sequencing chromatograms showing sequence variants identified in the *MEF2A* gene among the studied patients. **(A)** Homozygous *MEF2A* c.113826A > T substitution detected in patient P8 compared with the wild-type control sequence (C). **(B)** Homozygous *MEF2A* c.113802T > C substitution identified in patient P9 relative to the wild-type control sequence **(C)**. Homozygous variants are characterized by complete nucleotide replacement at the variant position compared with the corresponding wild-type sequence.

Mutations in the *MEF2A* gene were identified in one sample at nucleotide position 113826, where (**A**) adenine was replaced by (**T**) Thymine. In Fig 3A, “C” represents the wild-type (control), while P8, represent the mutations in patient 8 sample**.** Mutation in the *MEF2A* gene was identified in one sample at nucleotide position 113802, where (**T**) Thymine was replaced by (**C**) cytosine. In Fig 3B, “C” represents the wild-type (control), while P9, represent the mutations in the patient 9 sample**.**

### Gel electrophoresis

**Fig 4:**
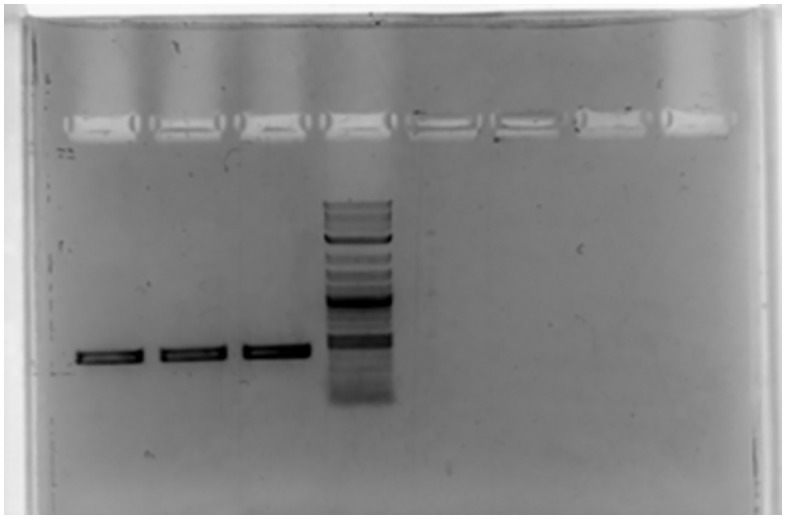
Agarose gel electrophoresis of PCR-amplified *MEF2A* gene fragments. Agarose gel electrophoresis showing PCR amplification of the *MEF2A* gene in the wild-type control and patient samples. Lane M, DNA molecular weight marker (DNA ladder); lane C, wild-type control; lane P8, patient carrying the homozygous *MEF2A* c.113826A > T substitution; and lane P9, patient carrying the homozygous *MEF2A* c.113802T > C substitution. Distinct bands at the expected amplicon size confirm successful amplification of the target *MEF2A* gene.

It shows the detection of *MEF2A* gene mutations by PCR analysis. Agarose gel electrophoresis showing PCR products of the *MEF2A* gene. The lane labeled C represents the wild-type (control) sample, while P8 and P9 represent patient samples. a nucleotide substitution was identified at position 113826, where adenine (A) was replaced by thymine (T) in patient P8. a nucleotide substitution was detected at position 113802, where thymine (T) was replaced by cytosine (C) in patient P9. The molecular weight marker (DNA ladder) was used to estimate fragment sizes. Differences in the electrophoretic banding patterns between control and patient samples confirm the presence of the respective *MEF2A* gene mutations. Details were also shown in Fig 4.

### Mutation in the *CYP27A1* gene

**Fig 5:**
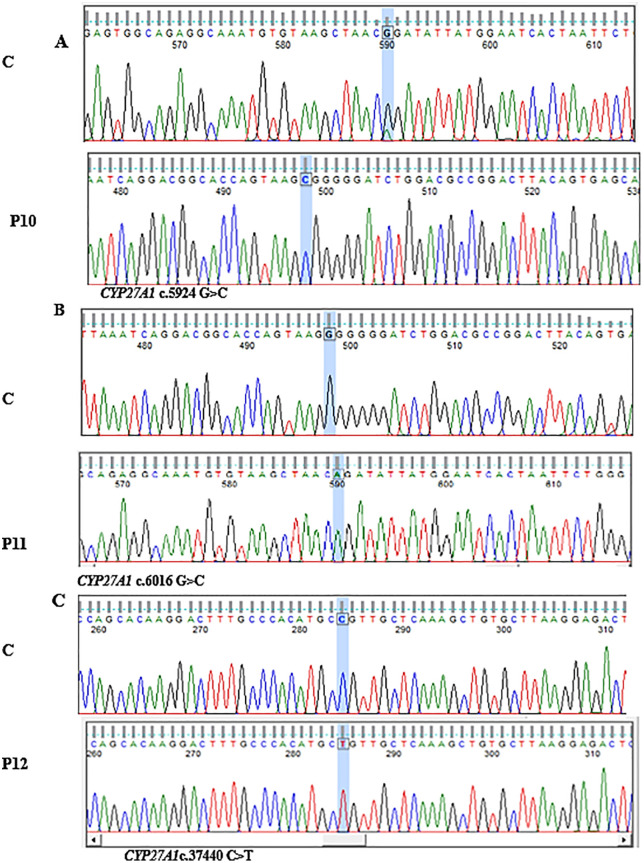
Sanger sequencing chromatograms of *CYP27A1* gene variants. Sanger sequencing chromatograms showing sequence variants identified in the *CYP27A1* gene among the studied patients. **(A)** Homozygous *CYP27A1* c.5924G > C substitution detected in patient P10 compared with the wild-type control sequence (C). **(B)** Homozygous *CYP27A1* c.6016G > C substitution identified in patient P11 relative to the wild-type control sequence (C). **(C)** Homozygous CYP27A1 c.37440C > T substitution identified in patient P12 compared with the corresponding wild-type control sequence (C). Homozygous variants are characterized by complete nucleotide replacement at the variant position compared with the corresponding wild-type sequence.

Mutation in the *CYP27A1* gene was identified in one sample of P10, at nucleotide position 5924, where (G) Guanine was replaced by (C) cytosine. In Fig 5A, “C” represents the wild-type (control), while P10, represent the mutations in the patient P10 sample. In Fig 5B “C” represents the wild-type (control), while mutation in the *CYP27A1* gene was identified in one sample of P11, at nucleotide position 6016, where (G) Guanine was replaced by (A) adenine. In Fig 5C, “C” represents the wild-type (control), while, represent the mutations in the patient (P11) sample. Mutation in the *CYP27A1* gene was identified in one sample of P12, at nucleotide position 37404, where (C) Cytosine was replaced by (T) Thymine. “C” represents the wild-type (control), while P12 represent the mutations in the patient (P12) sample.

### Gel electrophoresis

**Fig 6:**
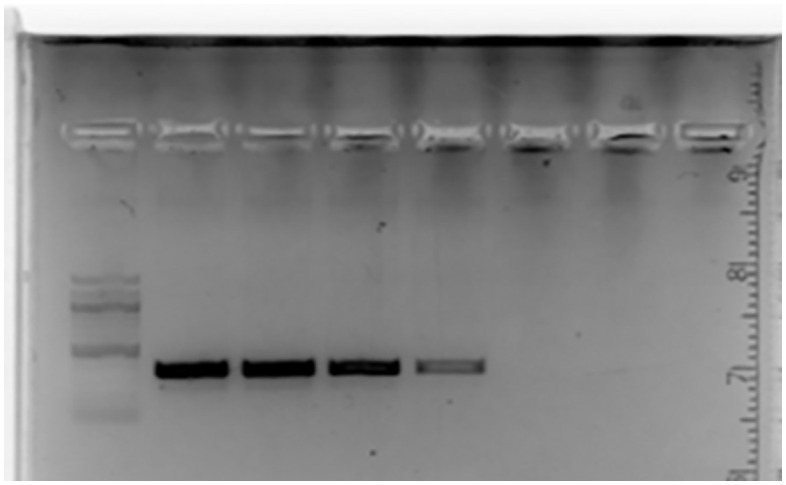
Agarose gel electrophoresis of PCR-amplified *CYP27A1* gene fragments. Gel electrophoresis showing PCR amplification of the *CYP27A1* gene in the wild-type control and patient samples. Lane M, DNA molecular weight marker (DNA ladder); lane C, wild-type control; lane P10, patient carrying the *CYP27A1* c.5924G > C substitution; lane P11, patient carrying the *CYP27A1* c.6016G > A substitution; and lane P12, patient carrying the *CYP27A1* c.37404C > T substitution. Distinct bands at the expected amplicon size confirm successful amplification of the target CYP27A1 gene.

Agarose gel electrophoresis showing PCR amplification of the *CYP27A1* gene fragment from control and patient samples. Lane M represents the DNA molecular weight marker. Lane C represents the wild-type (control) sample, while the patient lanes show amplified products from the respective samples. Distinct PCR bands of the expected size were observed in both control and patient samples, confirming successful amplification of the target region. Sequence analysis of the amplified products revealed nucleotide substitutions in the *CYP27A1* gene. In patient P10, a mutation was identified at nucleotide position 5924, where guanine (G) was replaced by cytosine (C). In patient P11, a mutation was detected at nucleotide position 6016, where guanine (G) was replaced by adenine (A). In patient P12, a mutation was identified at nucleotide position 37404, where cytosine (C) was replaced by thymine (T). details were also shown in Fig 6. Raw gel pictures were included in S1 Fig.

## Discussion

CVD is a leading cause of morbidity and mortality worldwide, with an increasing incidence among young individuals. The traditional risk factors such as hypertension, dyslipidemia, and smoking contribute significantly to CVD, genetic predisposition also plays an important role [36]. Among the genetic factors implicated, the *LRP6* gene has emerged as a key player in CVD pathogenesis. The *LRP6* gene encodes a co-receptor integral to the Wnt/β-catenin signaling pathway, which is essential for various physiological processes. Mutations in *LRP6* have been implicated in the pathogenesis of CVD, particularly in young individuals presenting with early-onset conditions. This mutation was associated with impaired Wnt signaling, suggesting a mechanistic link between *LRP6* dysfunction and increased CVD susceptibility [37]. Subsequent research has reinforced the association between *LRP6* mutations and CVD [38]. A study by Liyang et al. (2012) reported a novel mutation in the YWTD domain of *LRP6* that impaired endothelial cell proliferation and migration, processes vital for maintaining vascular integrity. This impairment was linked to familial CAD, underscoring the significance of *LRP6* mutations in hereditary forms of the disease [39]. Moreover, *LRP6*-mediated signaling pathways have been implicated in various cardiovascular conditions beyond CAD. Kang (2020) highlighted the involvement of *LRP6* in regulating blood pressure, glucose metabolism, and lipid homeostasis, all of which are critical factors in cardiovascular health. Dysregulation of these pathways due to *LRP6* mutations may contribute to the development of CVD in young individuals [40]. The mutations T146344A, A146345G, C92497A, and T94776A in the *LRP6* gene may have significant functional implications, particularly if they affect gene regulation or splicing. While their exact roles require further experimental validation, their potential association with diseases such as cancer, metabolic disorders, and skeletal abnormalities underscores the importance of *LRP6* in human health. Future studies should focus on elucidating the mechanisms by which these mutations contribute to disease pathogenesis and exploring therapeutic strategies targeting *LRP6* and the Wnt signaling pathway. In the current study, we also identified the following mutations in the *LRP6* gene in four patients. The specific nucleotide changes you’ve identified T146344A, A146345G, C92497A, and T94776A do not correspond to well-characterized mutations in the current literature. It’s essential to determine the exact nucleotide positions within the *LRP6* gene’s coding sequence to predict their potential impact on protein function. This involves identifying whether these mutations result in synonymous changes, missense mutations, or nonsense mutations, each of which can have varying effects on protein structure and function. For instance, missense mutations can lead to amino acid substitutions that may alter protein function. A study by Singh et al. (2013) identified a heterozygous missense mutation (c.1418G > A, p.Arg473Gln) in exon 7 of the *LRP6* gene, which was associated with early-onset coronary artery disease and other metabolic disorders [41]. Additionally, a novel heterozygous missense variant (c.719C > T, p.Thr240Ile) in exon 4 of the *LRP6* gene was identified in a family exhibiting high bone mass. This mutation did not affect gene transcription but impaired the maturation and phosphorylation of the *LRP6* protein, suggesting a disruption in Wnt signaling [42]. While the specific mutations T146344A, A146345G, C92497A, and T94776A in the *LRP6* gene are not well-documented in current literature, understanding their potential impact requires detailed genetic and functional analyses. Given the established role of *LRP6* in critical signaling pathways, mutations in this gene warrant thorough investigation to elucidate their clinical significance. The myocyte enhancer factor 2A (*MEF2A*) gene encodes a transcription factor integral to the regulation of gene expression in cardiovascular development and function. Its role in CVD, particularly in young individuals, has been a subject of research interest. Initial studies suggested a potential link between *MEF2A* mutations and CAD. A notable study identified a 21-base pair deletion in the *MEF2A* gene that appeared to co-segregate with CAD in a large family, implicating this mutation in the disease’s pathogenesis [43]. However, subsequent research challenged this association. For instance, a study by Pennacchio et al. sequenced *MEF2A* in approximately 300 patients with premature CAD and found no causative mutations. Moreover, the previously implicated 21-base pair deletion was also identified in individuals without CAD, suggesting that this specific mutation may not be pathogenic [44]. Further investigations have yielded mixed results. A study focusing on a Chinese Han pedigree identified a novel 6-base pair deletion in exon 11 of *MEF2A* that co-segregated with premature CAD/myocardial infarction (MI) within the family. This deletion was absent in sporadic cases and unrelated healthy controls, indicating a potential family-specific pathogenic variant [45,46] Functional studies have provided insights into the potential mechanisms by which *MEF2A* mutations could influence cardiovascular health. Research indicates that *MEF2A* plays a crucial role in endothelial cell function, and its deletion or mutation can lead to vascular endothelial dysfunction, a key factor in the development of CVD [47]. The impact of *MEF2A* mutations on cardiovascular health may be limited to particular genetic contexts, and further research is necessary to elucidate the precise role of *MEF2A* in CVD, especially among young individuals. A 21-base pair (bp) deletion in the *MEF2A* gene was first reported in a family with autosomal dominant CAD. This mutation was associated with impaired endothelial function and increased CAD risk in young individuals [48]. Subsequent studies have identified additional *MEF2A* variants (e.g., G283D, P279L) associated with early-onset CAD and endothelial dysfunction [28]. In this study the specific *MEF2A* mutations (A113826T, T113802C) were found in two patients. The A113826T mutation is located in a non-coding region of the *MEF2A* gene, potentially can affect the gene regulation, splicing, or mRNA stability. These non-coding mutations can influence transcriptional regulation or splicing efficiency. Non-coding mutations in *MEF2A* have been implicated in conditions such as CAD and endothelial dysfunction. Reduced *MEF2A* expression has been linked to impaired endothelial function and increased CVD risk in young individuals (Wang et al., 2003). The T113802C mutation is also in a non-coding region, possibly affecting regulatory elements or splicing. Similar to A113826T, this mutation may influence *MEF2A* expression or mRNA processing. *MEF2A* gene mutations, including A113826T and T113802C, are increasingly recognized as genetic risk factors for CVD in young individuals. These mutations may disrupt vascular development, endothelial function, and oxidative stress regulation, all of which contribute to early-onset CVD. Understanding the role of *MEF2A* in CVD pathogenesis provides valuable insights into the genetic basis of the disease and opens new avenues for early diagnosis, personalized therapy, and targeted interventions. Further research is needed to fully elucidate the mechanisms linking *MEF2A* mutations to CVD and to develop effective treatments for affected individuals. *CYP27A1* is a mitochondrial enzyme that plays a crucial role in cholesterol metabolism and bile acid synthesis. The *CYP27A1* gene encodes sterol 27-hydroxylase, a mitochondrial enzyme pivotal in bile acid synthesis and cholesterol homeostasis. It catalyzes the hydroxylation of cholesterol to form 27-hydroxycholesterol (27-OHC), an oxysterol that regulates lipid homeostasis, inflammation, and vascular function. In the cardiovascular system, *CYP27A1* is essential for maintaining cholesterol balance, endothelial function, and anti-inflammatory responses. *CYP27A1* converts cholesterol into 27-OHC, which can be further metabolized into bile acids. This process helps regulate cholesterol levels and prevent lipid accumulation in arterial walls [49]. 27-OHC has been shown to modulate endothelial function by influencing nitric oxide (NO) production and oxidative stress. Dysregulation of *CYP27A1* can lead to endothelial dysfunction, a key early event in atherosclerosis. *CYP27A1* and 27-OHC play a role in modulating immune responses and inflammation, which are central to CVD pathogenesis [50]. The specific nucleotide changes G6016A and C37484T in the *CYP27A1* gene correspond to mutations that have been identified in individuals with CTX. For instance, the c.1415G > C (p.Arg472Pro) mutation has been reported in multiple studies and is associated with the disease phenotype. This particular mutation leads to a substitution of arginine with proline at position 472, affecting the enzyme’s function. In our study we found mutation in 2 patients at position (G6016A, C37484T). This mutation (G6016A) is located in a non-coding region of the *CYP27A1* gene, potentially affecting gene regulation, splicing, or mRNA stability. These non-coding mutations can influence transcriptional regulation or splicing efficiency. For example, mutations in regulatory regions such as enhancers or silencers can alter *CYP27A1* expression levels, potentially disrupting cholesterol metabolism and endothelial function. The C37484T mutation is also in a non-coding region, possibly affecting regulatory elements or splicing. Similar to G6016A, this mutation may influence *CYP27A1* expression or mRNA processing. Computational tools like Ensembl’s Variant Effect Predictor (VEP) can provide insights into its potential impact on regulatory motifs. The non-coding variants in *CYP27A1* have been associated with metabolic syndromes, including type 2 diabetes and hyperlipidemia, due to their role in lipid metabolism and insulin signaling.

## Conclusion

This study identified multiple genetic variants in *LRP6*, *MEF2A*, and *CYP27A1* genes among familial CVD patients in a high-consanguinity population. These variants were observed in a substantial proportion of cases, suggesting a potential genetic contribution to early-onset cardiovascular disease in this region. Our findings highlight the importance of incorporating genetic screening strategies for early detection and risk assessment in high-risk populations. The presence of multiple mutations in these genes suggests their potential involvement in the pathogenesis of CVD, warranting further investigation into their functional implications. The increasing prevalence of CVD in younger populations is a pressing concern, particularly in regions with high rates of familial predisposition and consanguineous marriages. Such genetic factors may exacerbate disease risk by increasing the likelihood of inheriting pathogenic mutations. The inclusion of *ST6GALNAC5*, another gene of interest, underscores the complexity of genetic contributions to CVD, emphasizing the need for a broader genomic perspective in future studies. A multidisciplinary approach involving geneticists, cardiologists, and policymakers is essential to develop effective prevention and management programs tailored to the genetic profile of this population. This study highlights the important contribution of genetic factors to cardiovascular disease risk among young adults in Khyber Pakhtunkhwa, with pathogenic variants identified in more than half of the studied patients, particularly in the *LRP6, MEF2A, and CYP27A1* genes. The increasing burden of cardiovascular disease among younger individuals is especially concerning in settings where familial clustering and consanguineous marriages are common, as these practices may increase the transmission of harmful genetic variants. Incorporating genetic evidence into national cardiovascular health policies may help reduce the long-term burden of disease and improve outcomes for young adults in resource limited settings. Future studies employing larger cohorts and advanced sequencing technologies are recommended to validate these findings and further elucidate the genetic architecture of cardiovascular disease in this population.

## Supporting information

S1 Fig Agarose gel electrophoresis of PCR-amplified target gene fragments. Agarose gel electrophoresis demonstrating successful PCR amplification of the **(A)** *LRP6*, **(B)** *CYP27A1*, and **(C)** *MEF2A* gene fragments. Distinct bands corresponding to the expected amplicon sizes were observed in the sample lanes, confirming successful amplification of the target genes. Lane M represents the DNA molecular weight marker (DNA ladder).
(PDF)

S1 File Graphical abstract. (TIF)
