# Supplementary material for: Targeted sequencing provides genetic insights into familial cardiovascular disease among young adults in a high consanguinity population
Source: PLoS One. 2026 Aug 13;21(8):e0353861. doi: 10.1371/journal.pone.0353861 (PMC13472394; doi:10.1371/journal.pone.0353861)
Supplement: Supplementary file 1 [file pone.0353861.s001.pdf]

(A)

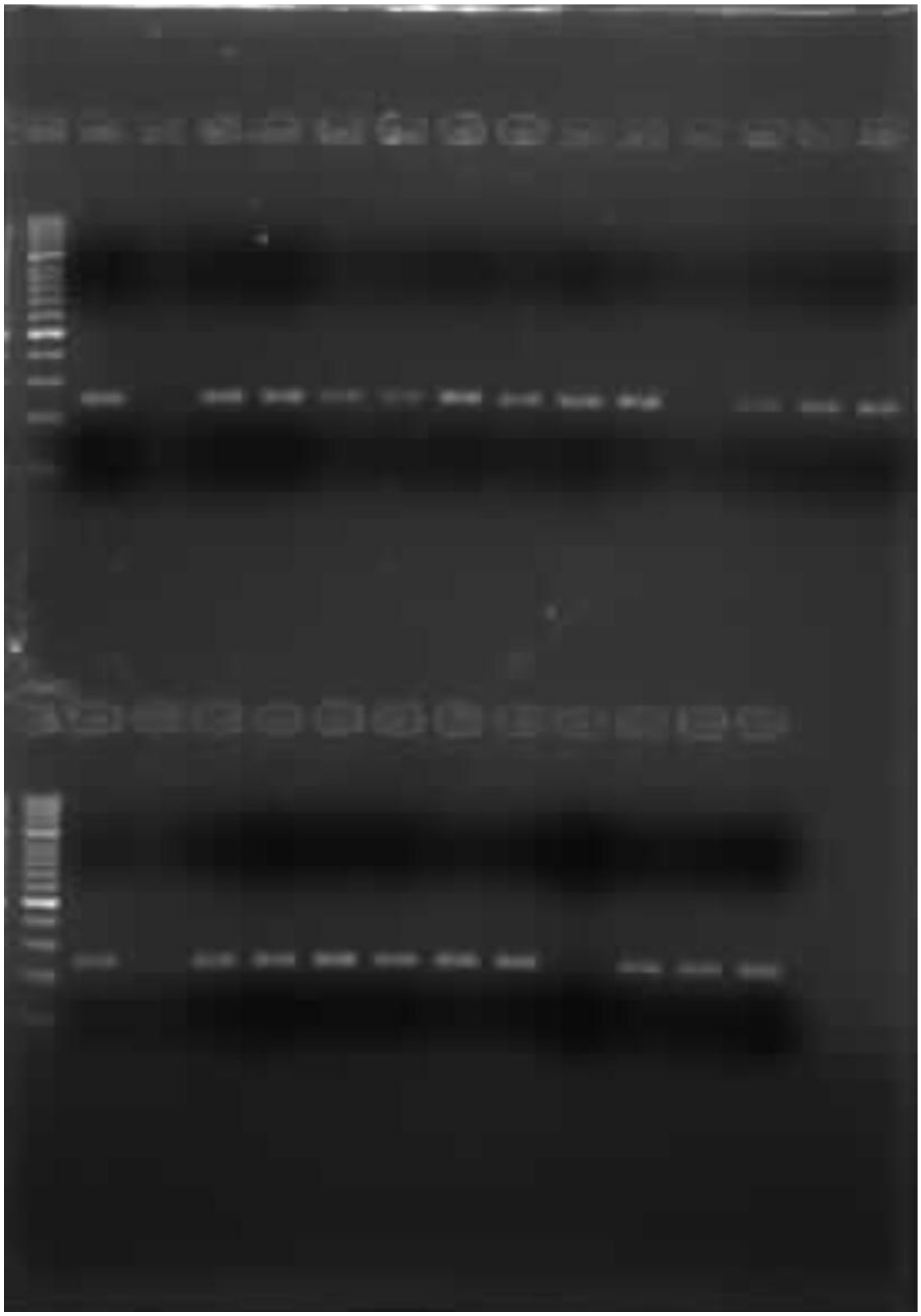

(B)

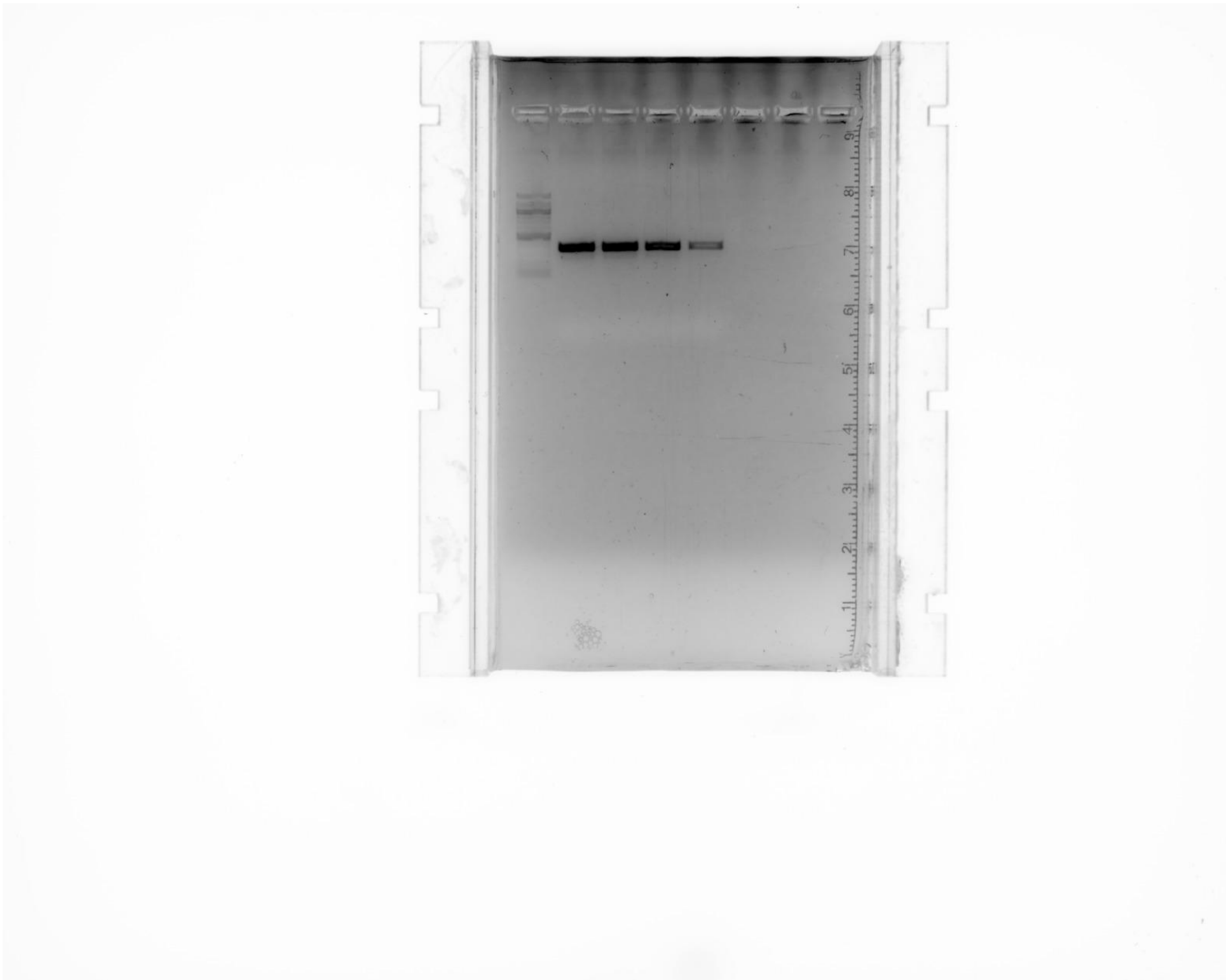

(C)

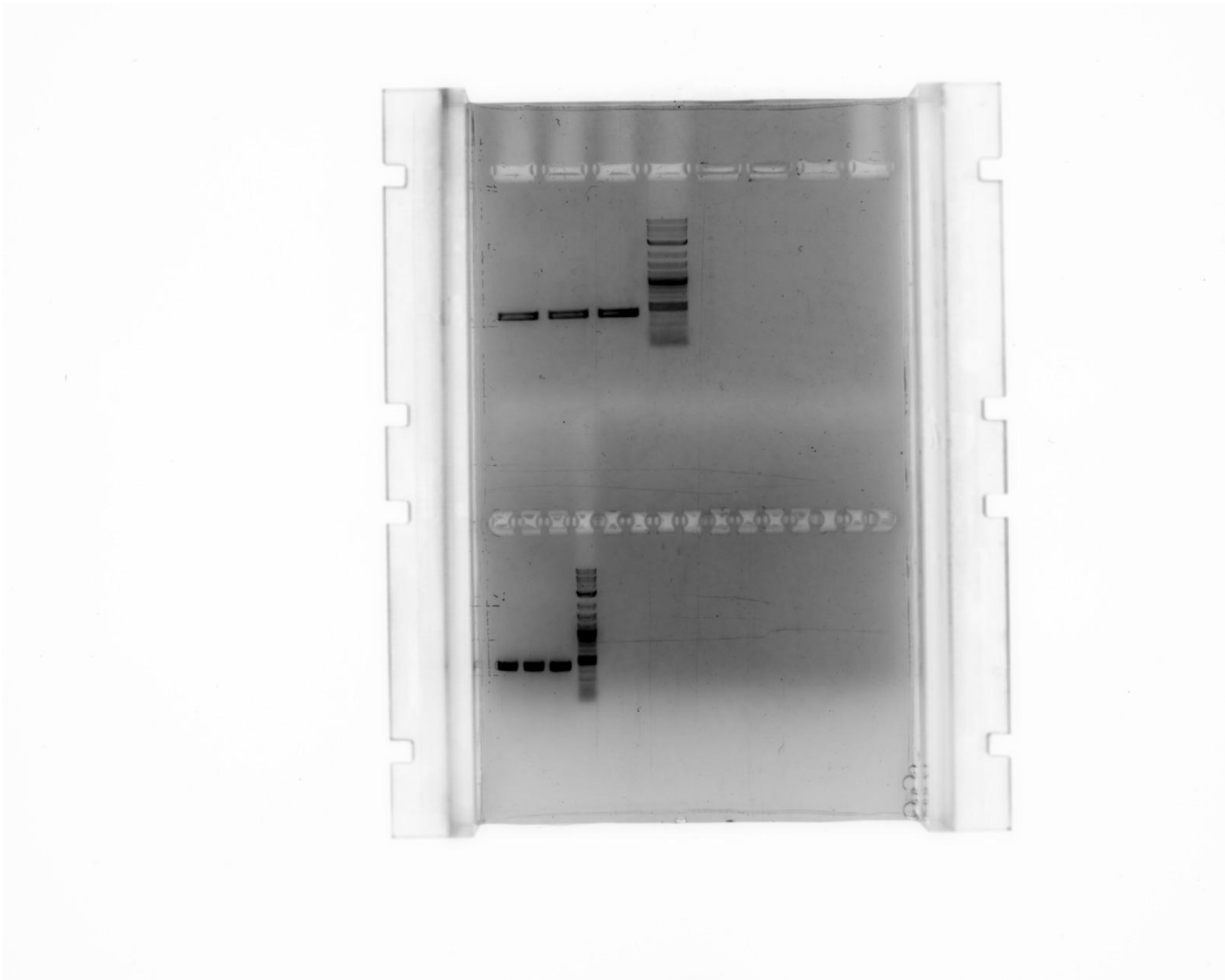

S1 Fig. Agarose gel electrophoresis of PCR-amplified target gene fragments. Agarose gel electrophoresis demonstrating successful PCR amplification of the **(A)** *LRP6*, **(B)** *CYP27A1*, and **(C)** *MEF2A* gene fragments. Distinct bands corresponding to the expected amplicon sizes were observed in the sample lanes, confirming successful amplification of the target genes. Lane M represents the DNA molecular weight marker (DNA ladder).
